# Characterization of *Syzygium cumini* (L.) Skeels (Jamun Seed) Particulate Fillers for Their Potential Use in Polymer Composites

**DOI:** 10.3390/molecules29112618

**Published:** 2024-06-02

**Authors:** Senthil Muthu Kumar Thiagamani, Chalasani Venkata Yaswanth, Chaganti Yashwanth, Thanh Mai Nguyen Tran, Senthilkumar Krishnasamy, Muthukumaran Azhaguchamy, Anish Khan, Mohamed Hashem, Hassan Fouad

**Affiliations:** 1 Department of Mechanical Engineering, Kalasalingam Academy of Research and Education, Anand Nagar, Krishnankoil 626126, Tamil Nadu, India; 2 Department of Mechanical Engineering, INTI International University, Persiaran Perdana BBN, Putra Nilai, Nilai 71800, Negeri Sembilan, Malaysia; anishkhan97@gmail.com; 3 Centre for Advanced Composite Materials (CACM) Universiti Teknologi Malaysia, Skudai, Johor Bahru 81310, Johor, Malaysia; 4 Department of Automobile Engineering, Kalasalingam Academy of Research and Education, Anand Nagar, Krishnankoil 626126, Tamil Nadu, India; 5 Department of Transportation Construction, Faculty of Civil Engineering, Nha Trang University, 02 Nguyen Dinh Chieu St., Nha Trang 650000, Vietnam; thanhmnt@ntu.edu.vn; 6 Department of Mechanical Engineering, PSG Institute of Technology and Applied Research, Coimbatore, Neelambur 641062, Tamil Nadu, India; kmsenthilkumar@gmail.com; 7 Department of Biotechnology, Kalasalingam Academy of Research and Education, Anand Nagar, Krishnankoil 626126, Tamil Nadu, India; a.muthukumaran@klu.ac.in; 8 Department of Dental Health, College of Applied Medical Sciences, King Saud University, Riyadh P.O. Box 12372, Saudi Arabia; mihashem@ksu.edu.sa; 9 Applied Medical Science Department, Community College, King Saud University, Riyadh P.O. Box 11433, Saudi Arabia; menhfef@ksu.edu.sa

**Keywords:** *Syzgium cumini* (L.) Skeels powder, physicochemical, scanning electron microscope, crystallinity index, thermogravimetric analysis, differential scanning calorimeter, antioxidant

## Abstract

*Syzgium cumini* (L.) Skeels powder (*S. cumini* powder), also known as Jamun, is well-known for its various medical and health benefits. It is especially recognized for its antidiabetic and antioxidant properties. Thus, *S. cumini* powder is used in various industries, such as the food and cosmetic industries. In this work, the fruit of *S. cumini* was utilized; its seeds were extracted, dried, and ground into powder. The ground powders were subjected to various techniques such as physicochemical tests, Fourier transform infrared (FTIR) spectroscopy, X-ray diffractometry (XRD), particle size analysis, scanning electron microscopy (SEM), energy-dispersive X-ray spectroscopy (EDX), thermogravimetric analysis (TGA), differential scanning calorimetry (DSC), and antioxidant analysis. From the physicochemical tests, it was revealed that the jamun seed filler contained cellulose (43.28%), hemicellulose (19.88%), lignin (23.28%), pectin (12.58%), and wax (0.98%). The FTIR analysis supported these results. For instance, a peak at 2889 cm^−1^ was observed and associated with CH stretching, typically found in methyl and methylene groups, characteristic of cellulose and hemicellulose structures. The XRD results demonstrated that the crystallinity index of the jamun seed filler was 42.63%. The particle analysis indicated that the mean (average) particle size was 25.34 μm. This observation was ensured with SEM results. The EDX spectrum results showed the elemental composition of the fillers. Regarding thermal degradation, the jamun seed filler had the ability to withstand temperatures of up to 316.5 °C. Furthermore, endothermic and exothermic peaks were observed at 305 °C and 400 °C, respectively. Furthermore, the antioxidant property of the powder displayed a peak scavenging activity of 91.4%. This comprehensive study not only underscores the viability of *S. cumini* powder as a sustainable and effective particulate filler in polymer composites but also demonstrates its potential to enhance the mechanical properties of composites, thereby offering significant implications for the development of eco-friendly materials in various industrial applications.

## 1. Introduction

A global shift towards sustainability is driving the increasing adoption of natural materials across various industries. Governments worldwide have been advocating for the utilization of natural materials to minimize environmental impact, and research has demonstrated the substantial benefits of these materials over synthetic alternatives. Natural materials, such as plant fibers, are readily available and environmentally friendly. They also offer advantages such as a lower density and simpler processing methods, making them highly suitable for a wide range of applications [1,2,3]. 

In reinforcing materials, natural fibers and fillers are extensively studied for their potential to enhance the properties of different matrices. These materials have found applications across diverse sectors, including the automotive, construction, biomedical, electronics, and sports sectors [3,4,5]. For instance, major automotive manufacturers like Mercedes-Benz [6], Audi A2 [7], Toyota [8], Mitsubishi Motors [8] and BMW [9] have successfully integrated natural fillers with various matrices to produce components such as door panels and interior elements. This integration reduces the environmental footprint and leverages the mechanical advantages of natural fillers. 

The following Table 1 summarizes key studies on different natural fibers, highlighting their composition and properties that align with the applications explored in our study:

The inclusion of these comparisons underscores the relevance of natural fibers like those derived from *Syzygium cumini* (L.) Skeels, which is the focus of our study. The physicochemical properties of *S. cumini* seeds, particularly their cellulose, hemicellulose, and lignin content, along with their antioxidant potential, make them promising candidates as reinforcing fillers in polymer composites. This study aims to explore these characteristics further and evaluate the practical applications of *S. cumini* seed powder in various industries. 

Antioxidants are essential compounds that counteract harmful molecules known as free radicals in the body, which are linked to various chronic diseases such as cancer, diabetes, and cardiovascular ailments [17]. Thus, incorporating antioxidant-rich foods like *S. cumini* powder into one’s diet may help to combat oxidative stress and enhance overall health. The antioxidant potential of *S. cumini* powder is crucial for its nutritional value and potential therapeutic applications [18]. *S. cumini* seeds contain bioactive compounds like polyphenols, flavonoids, and tannins, which exhibit antioxidant properties by scavenging free radicals, thereby protecting cells from damage and lowering the risk of chronic diseases. Furthermore, research indicates that *S. cumini* powder demonstrates significant radical scavenging activity, making it a promising natural source of antioxidants [19,20]. 

*Syzygium cumini* (L.) Skeels is a tree in the *Myrtaceae* family that originated in India and is now widely dispersed throughout Asia, including Malaysia, Thailand and the Philippines. The fruits are elliptically shaped juicy berries with a solitary dark brown seed in the center. Its diameter is about 2 cm, and its length ranges from 1.5 to 3.5 cm. The fruits are distributed throughout the tree canopy in clusters [17,18,19]. The main chemical components of the fruit and seed are anthocyanins (found in the pulp) and other phenolics. Additionally mentioned were calcium, protein, phenolics, and flavonoids [18,19]. Ayurvedic medicine claims that *S. cumini* seeds, leaves, and stem bark can treat diabetes [19]. Beyond these applications, *S. cumini* seeds are usually thrown away as waste since they are not very useful for direct food intake or replanting. These seeds are given new life as useful fillers in composite materials [20], improving resource utilization and sustainability across a range of industries. This creative method increases the economic and ecological effectiveness of these seeds while also decreasing waste. 

Based on a detailed literature review, it was observed that the preparation and characterization of *S. cumini* powder have not been examined in earlier studies. Thus, in this present work, the authors explored its characteristics using various techniques such as physicochemical, FTIR, XRD, particle size analysis, SEM, TGA, and DSC. Based on the observed results, the authors concluded that *S. cumini* powder could be used as a reinforcement for fabricating green composites and utilized for structural-based applications in various industries. 

## 2. Results and Discussions

### 2.1. Physicochemical Analysis

The physicochemical composition of *S. cumini* powder was quantitatively analyzed and is detailed in Table 2, revealing a diverse distribution of components. The primary constituent is cellulose, accounting for 43.28% by weight. Cellulose, a homopolysaccharide comprising glucose units, is the predominant structural element in the seed powder. Although this percentage is lower compared to some natural fillers such as peanut shells and certain sunflower seed hull fillers, it exceeds the cellulose content found in other agricultural fillers like rice husks, walnut shells, coconut shells, and soybean hulls [21,22]. This comparative analysis suggests that the mechanical and thermal properties of polymer composites incorporating *S. cumini* seed powder might surpass those of composites using other agro-based fillers [22]. Hemicellulose comprises 20.08% of the *S. cumini* seed powder by weight, aligning with the levels typically found in other nutshells but lower than those in walnut shell and soybean hulls, and higher than those in peanut shell fillers [22]. Hemicellulose, known for its impact on water absorption, biodegradation, and thermal stability, degrades at lower temperatures than cellulose, influencing the overall durability and stability of the composites [23]. Lignin, an amorphous polymer, comprises 23.28% of the *S. cumini* powder. Its presence is beneficial for enhancing the compatibility with matrix materials and providing a degree of flame retardancy, which is advantageous for composites used in building materials. Additionally, the filler contains 12.58% pectin, which plays a role in the water absorption, adhesion, biodegradation, and flexibility of the composites [24]. The low wax content, at 0.98%, though significant, is lower than that found in rice husk and comparable to levels in PVNS fillers, which could enhance the bonding with matrix materials [25,26]. Moisture content is reported at 11.53%, a factor that could influence porosity, dimensional stability, mechanical properties, and the swelling behavior of the polymer composites [25]. Lastly, an ash content of 10.76% could impact the fire-resistant properties and contribute to the elimination of amorphous elements in the composites [26]. 

### 2.2. FTIR Analysis

**Figure 1:**
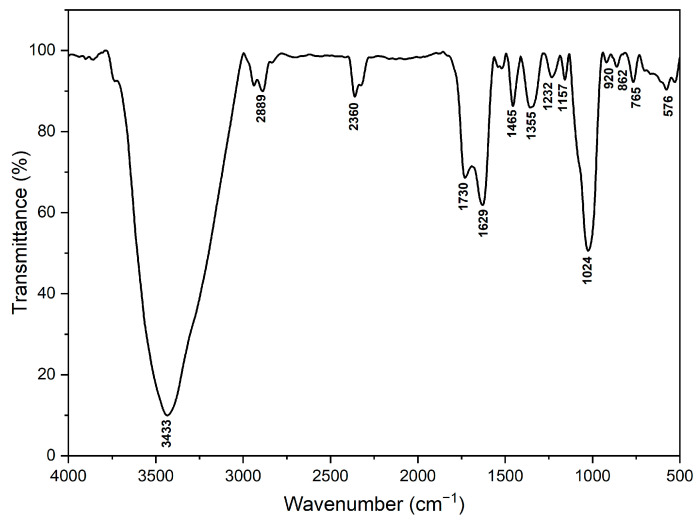
FTIR spectra of *S. cumini* powder.

The FT-IR analysis of raw *S. cumini* [34,35] reveals distinct absorption bands corresponding to various chemical functional groups inherent in cellulose, hemicellulose, and lignin, as shown in the spectrum ranging from 4000 to 500 cm^−1^ (Figure 1 and Table 2). Several key peaks are observed, each indicative of specific molecular interactions and structures. At 3433 cm^−1^, a prominent peak is observed, attributed to moisture or hydroxyl groups within cellulose. This peak indicates hydrogen-bonded O-H groups, with its intensity and shape offering insights into the hydrogen bonding network within cellulose and its interactions with water molecules [36,37,38,39]. The peak at 2889 cm^−1^ is associated with CH stretching, typically found in methyl and methylene groups, characteristic of cellulose and hemicellulose structures [36,37,38,39]. Additionally, the peak at 2360 cm^−1^, although potentially indicating atmospheric CO_2_ or the presence of triple bonds, might not be directly related to the primary components of the Jamun seed filler. Instead, this peak may arise from impurities or environmental factors [40]. A sharp peak at 1730 cm^−1^ indicates C=O stretching in the lignin and hemicellulose fractions, suggesting the presence of carbonyl groups within the JS. This feature is crucial for understanding the compositional and structural properties of the seed filler [36,41,42]. The peak at 1629 cm^−1^ is associated with water absorption, likely due to water molecules absorbed in the sample, as cellulose and hemicellulose are known to form intermolecular hydrogen bonds with water [41,43,44]. Meanwhile, the peak at 1465 cm^−1^ is attributed to CH_3_ deformation asymmetry in lignin and CH_2_ bending, pointing towards the presence of lignin and xylan [36,38,45]. An extensive peak at 1355 cm^−1^ correlates with C-H bending vibrations, essential for discerning the molecular structure and interactions within the biomass [36,42,44]. At 1232 cm^−1^, a peak signifies C-O stretching in lignin, indicative of ether and phenolic structures, pivotal for comprehending the chemical structure of lignin and its potential applications [41,46,47]. The peaks at 1157 and 1024 cm^−1^ are indicative of the C-O-C pyranose ring structure in cellulose and hemicellulose, providing vital information about the polysaccharide backbone and its conformational aspects [38,42,48]. Lastly, the peaks at 920 and 862 cm^−1^ are attributed to the β-glycosidic linkages between glucose units in cellulose, which are crucial for understanding the structural integrity and mechanical properties of cellulose [44,46,49]. Table 3 provides the peak positions and allocations of chemical stretching in the *S. cumini* powder.

### 2.3. XRD Analysis

**Figure 2:**
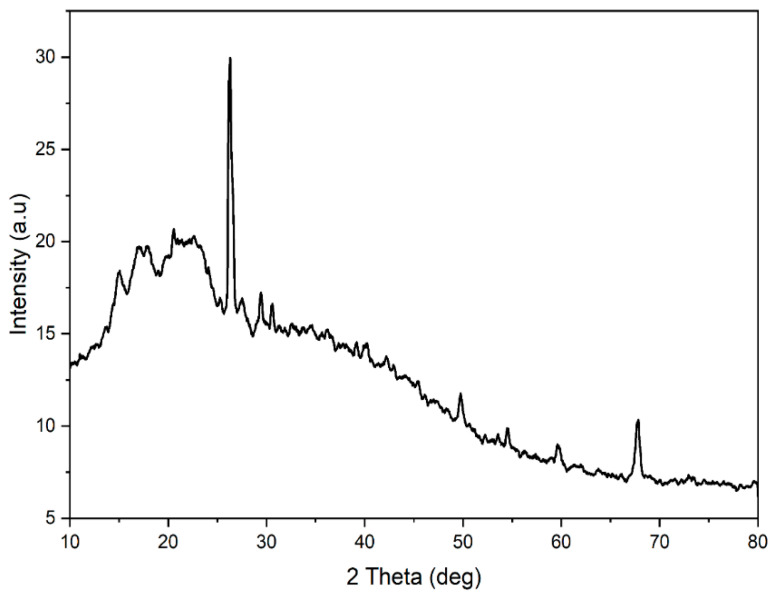
X-ray diffractogram of the *S. cumini* power.

The XRD analysis of *S. cumini* revealed (Figure 2) distinct crystalline peaks at 2θ diffraction angles of 15°, 20.54°, 24.14°, 26.25°, 26.5°, 29.44°, 30.56°, 49.74°, 54.53°, 67.75°, and 67.90°. These peaks correspond to cellulose types I and IV, as confirmed by complementary FT-IR analysis [50,51]. This alignment of XRD results with FT-IR analysis indicates the presence of both cellulose types I and IV in *S. cumini*. The crystallinity index (CI) of *S. cumini* was calculated to be 42.63%, indicating the degree of crystallinity relative to its amorphous content. This CI value is lower compared to other natural fibers such as *Lygeum spartum* L. (46.19%), *Sansevieria cylindrica* (60%), *Acacia planifrons* (65.38%), and *Cissus quadrangularis* root (56.6%) [50,52,53]. For reference, the CI values of various other natural fibers are reported as *Wrightia tinctoria* seed fiber (49.2%), *Ramie* fiber (58%), *Sansevieria cylindrica* leaf fibers (60%), *Raffia textilis* (64%), sisal (71%), jute (71%), flax (80%), and hemp (88%) [51].

Furthermore, the crystallite size of *S. cumini* was estimated to be 0.398 nm, which is notably smaller than that of *Prosopis juliflora* (15 nm), Cissus quadrangularis Vapor (31.55 nm), Cissus quadrangularis root (2.4 nm), and *Ferula communis* (1.6 nm) [54,55,56]. It is important to note that larger crystal size arrangements in natural fibers typically result in decreased chemical reactivity and water absorptivity. For context, the crystallite sizes of some other natural fibers are reported as follows: *Cissus quadrangularis* root (28.05 nm), *Sansevieria cylindrica* (86 nm), *Prosopis juliflora* (15 nm) [57]. *R. textilis* (32 nm), ramie fibers (16 nm), cotton fibers (5.5 nm), corn stalk fibers (3.8 nm), and flax fibers (2.8 nm) [51,58].

### 2.4. Particulate Size Evaluation

**Figure 3:**
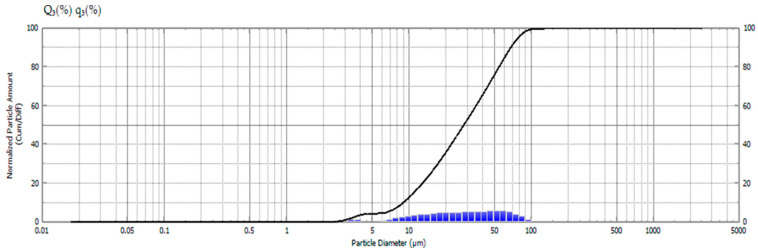
Particle size analysis of *S. cumini* powder.

In our study, the particle size distribution of *Syzygium cumini* seed powder was meticulously analyzed to understand its implications for potential applications in polymer composites. The results of this analysis are illustrated in Figure 3. The particle sizes of *S. cumini* powder were found to range between 1.98 and 127.66 μm, with a mean size of 25.34 μm. Notably, the mode, or the most frequently occurring particle size, was observed at 49.28 μm. This size distribution is significant as it influences the physical properties of the composites, such as density, porosity, and surface area, which in turn affect the mechanical strength and stability of the final product. This evaluation was intended to characterize the particle sizes resulting from the grinding process of the dried seeds. It was not aimed at assessing any treatment effects on the seeds themselves but rather the characteristics of the ground material that could be relevant for its application as a filler in polymer matrices.

### 2.5. Morphological Analysis

**Figure 4:**
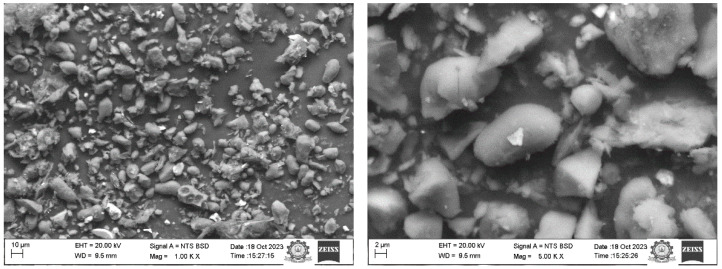
SEM micrographs of *S. cumini* powder.

The surface morphology of *S. cumini* fillers was meticulously examined using scanning electron microscopy (SEM) at various magnifications, as depicted in Figure 4. Investigating the surface morphology is crucial for determining the potential of these particles as effective reinforcement agents. The SEM images in Figure 4 reveal that the diameter range of *S. cumini* particles varies between approximately 1 and 40 μm. The analysis through SEM supports the findings of the particle size analysis. The SEM images display particles characterized by a mix of irregular and smooth shapes in diverse sizes. Furthermore, Figure 4 also highlights the presence of some impurities on the surfaces of the particles. To enhance the bonding strength with polymer matrices and ensure a higher quality of the final composite material, it is often necessary to subject natural fillers to chemical treatments to eliminate these surface impurities.

**Figure 5:**
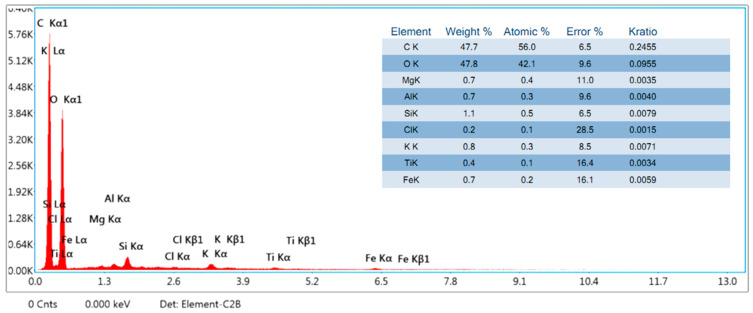
EDX spectra of the *S. cumini* powder.

The EDX analysis of *S. cumini* is illustrated in Figure 5. The EDX spectrum reveals the elemental composition of the fillers, predominantly carbon (C) and oxygen (O). This observation confirms the presence of these fundamental elements in the *S. cumini* particles. Additionally, the analysis detected the presence of magnesium (Mg), silicon (Si), aluminum (Al), iron (Fe), and potassium (K). The detection of these elements suggests the presence of various impurities within the *S. cumini* material, indicating that the raw *S. cumini* particles are not entirely pure.

### 2.6. Thermal Analysis

**Figure 6:**
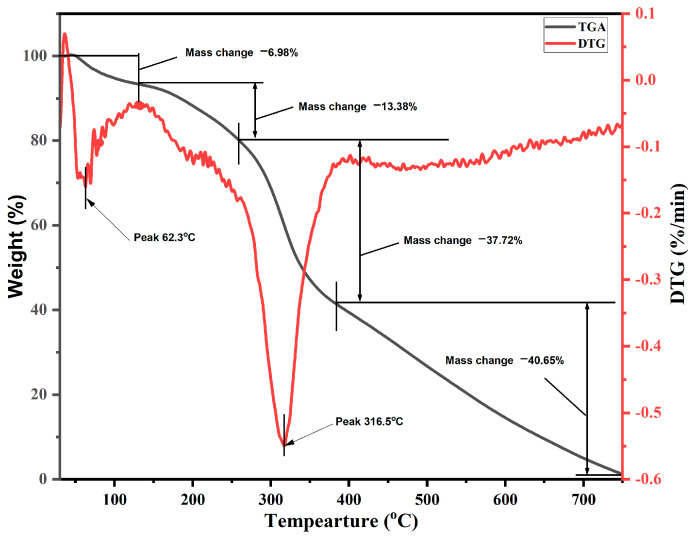
TGA/DTG thermograms of *S. cumini* powder.

The primary and derivative curves of *S. cumini* are illustrated in Figure 6. The TGA data indicate a multi-stage degradation process. In the first phase, degradation occurs from room temperature to 136 °C, attributed primarily to the loss of moisture with a mass loss of approximately 6.98%. The second phase, occurring between 136 °C and 262 °C, is characterized by a mass reduction of 13.38%. This loss is associated with the decomposition of hemicellulose and the glycosidic linkages in cellulose [57]. Subsequently, the complete decomposition of cellulose and the maximal release of volatile materials are observed between 262 °C and 380 °C, corresponding to a mass loss of 37.72% [59]. Beyond 380 °C, the residual mass is primarily char. In the DTG analysis, a significant peak at 316.5 °C is evident, indicative of the thermal degradation of cellulose I and the extensive degradation of α-cellulose. Comparative analysis shows similar peaks in various other natural fibers: *Lygeum spartum* at 338.7 °C, hemp at 308.2 °C, *Cissus quadrangularis* root at 328.9 °C, *Aristida hystrix* leaf fiber at 298.2 °C, and jute at 298.8 °C [52,53,60]. These results confirm that *S. cumini* is a viable candidate for composite applications reinforcement withstanding working temperatures up to 316.5 °C.

**Figure 7:**
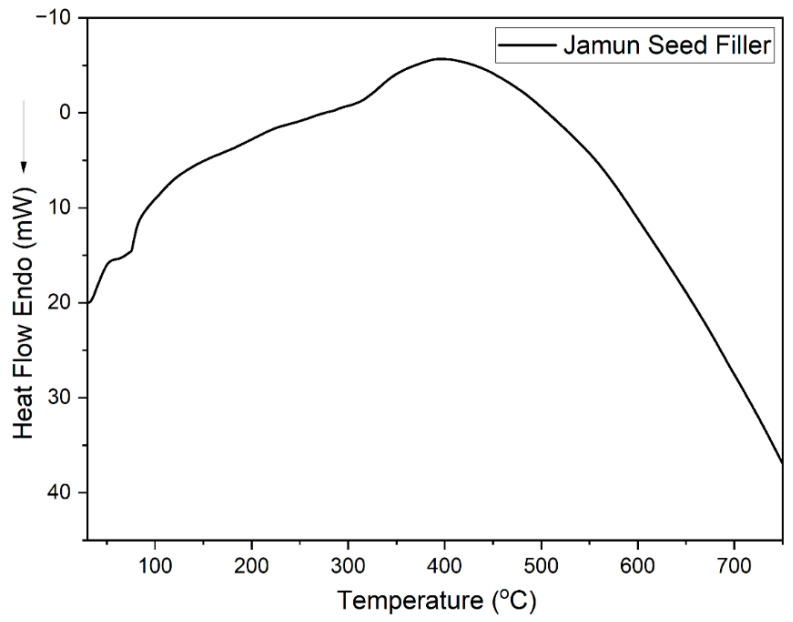
DSC thermograms of *S. cumini* powder.

The differential scanning calorimetry (DSC) curve for *S. cumini* fillers, as shown in Figure 7, supplements our TGA findings by providing additional insights into the thermal transitions of the material. The DSC analysis identifies an initial endothermic peak at approximately 305 °C, which correlates with the loss of bound moisture and the onset of hemicellulose degradation. This is followed by a broad exothermic peak around 400 °C, indicative of the extensive degradation of both the lignin and cellulose components present in the *S. cumini* particles.

While DSC provides valuable information about the energy absorption and release during these thermal transitions, it is important to note that the DSC is not used in isolation to study the degradation process. Instead, it complements the TGA data, which directly measure the mass loss associated with these thermal events. The consistency of this thermal decomposition pattern with those observed in similar natural materials, such as almond and poplar shells, supports the reliability of our analytical approach and corroborates our findings regarding the thermal stability of *S. cumini* particles [22].

### 2.7. Antioxidant Activity

DPPH activity is a primary method for evaluating the antioxidant properties of natural products. DPPH can be stabilized by accepting an electron, forming a stable molecule. Scavenging efficacy was determined by the decrease in absorbance at 517 nm, indicating the reduction of DPPH to its yellow form. The strong 517 nm absorption band in the purple methanolic solution suggests the presence of an unpaired electron [61]. In this study, we aimed to investigate the antioxidant activity of *S. cumini* powder. The *S. cumini* powder exhibited significant antioxidant activity, at 91.4%. These findings align with previous research [62] which demonstrated the antioxidant capacity of *S. cumini* powder to be 93.4%, 92.5%, and 96.2% under shade, sun, and freeze-dried conditions, respectively. In a separate study, the antioxidant activity of supplemented samples was found to be lowest in control juice (68.94%), highest in freeze-dried *S. cumini* powder supplemented with pear juice (82.63%), and second highest in sun-dried jamun powder supplemented with pear juice (80.26%) [63]. Ahmed et al. reported that *S. cumini* extracts of seed and pulp at a concentration of 1000 µg/mL could scavenge 96.84% and 94.97% of DPPH free radicals, respectively, while the standard compound ascorbic acid exhibited 98.35% DPPH free radical scavenging activity [64]. In another study, Babbar et al. analyzed the antioxidant activity of kinnow, litchi, and grape seed extracts, finding DPPH free radical scavenging activities of 67%, 83%, and 77%, respectively [65]. In the present study, the higher antioxidant activity of *S. cumini* may be due to the presence of phenolic content in the seed, as in the study conducted by Priya et al. Their research explored the antioxidant, phenolic-flavonoid content of three different variant of *S. cumini* seeds [61]. 

## 3. Materials and Methods

### 3.1. Materials

**Figure 8:**
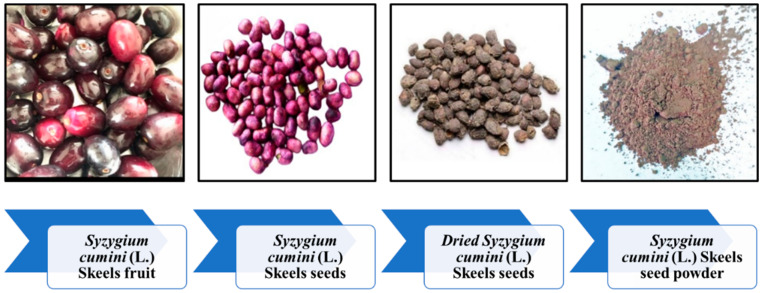
Process indicating the preparation of *S. cumini* powder.

After being extracted from the fruit, the *S. cumini* seeds were dried in the sun for approximately a week before being ground into a fine powder in a ball mill. The powder was then subjected to various characterizations to know the physiochemical contents, particle size, functional groups, structural and thermal behavior. The process involved in the preparation of the seed powder is shown in Figure 8. 

2,2-diphenyl-1-picrylhydrazyl were purchased from Himedia Laboratories Pvt. Ltd., India. MilliQ RO water (Milli-Q IQ 7003/05/10/15 Ultrapure & Pure Lab Water Purification System) was prepared in the laboratory. 

### 3.2. Characterization Techniques

#### 3.2.1. Physiochemical Analysis

The physiochemical examination of *S. cumini* powder was performed to determine the weight percentages of several components, including cellulose, hemicellulose, lignin, pectin, wax, and moisture. The density of the filler was also determined during the investigation. Standard procedures were followed for this evaluation per our preceding reports [66,67,68]. 

#### 3.2.2. Fourier Transform Infrared (FTIR) Spectroscopy Analysis

The FTIR analysis of *S. cumini* powder was performed using an IR Tracer 100 spectrometer. (Shimadzu Corporation, Tokyo, Japan) Samples were prepared by mixing the powdered material with potassium bromide (KBr) at a 1:100 ratio and compressing into pellets. The analysis was conducted under ambient conditions. Each sample was scanned over a range of 4000 to 400 cm^−1^ with a resolution of 4 cm^−1^. A total of 32 scans were averaged for each sample to ensure accuracy and reduce noise in the spectral data.

#### 3.2.3. X-Ray Diffraction (XRD) Analysis

The structural properties of the *S. cumini* powder were examined by a D8 Advance ECO X-ray diffractometer in the 2-theta range of 10–80°. Scanning was performed at 4°/min. 

#### 3.2.4. Particle Size Evaluation

The particle size of the *S. cumini* powder was measured using a Shimadzu SALD-2300 (V 3.1.1) particle size analyzer. Each 100 particles had their size assessed in a variety of ranges.

#### 3.2.5. SEM Analysis

The morphology of the power was recorded using a scanning electron microscope (EVO18, CARL ZEISS). (Oberkochen, Germany) Various electron wavelengths, ranging from 100 to 3000, were used to scan the filler with an accelerated voltage of 10 kV, facilitating varying magnified levels. 

#### 3.2.6. Thermogravimetric Analysis (TGA)

A TGA Q500, (TA Instruments, Lukens Drive New Castle, DE 19720, USA) machine was used to record the TG curves of the *S. cumini* powder throughout a temperature range of 30 to 700 °C at a rate of 10 °C/min. The analysis was performed in a nitrogen-filled atmosphere at a 60 mL/min flow rate.

#### 3.2.7. Antioxidant Property

In the present study, the method previously reported by Naveenkumar et al. was adapted to assess the antioxidant property of *S. cumini* powder [69]. The study was carried out using a UV-vis spectrometer (SHIMADZU UV-1800, Tokyo, Japan) at a wavelength of 517 nm. DPPH (0.1 mM) was prepared and dissolved in methanol. Followed by this, 1 mL of *S. cumini* powder (0.1 µg/mL) and 3 mL of DPPH solution were combined, and the combination was left to incubate in the dark for 30 min. DPPH solution was utilized as a control, and absorbance was measured at 517 nm. Equation (1) was used to observe the degradation of DPPH.

(1) $$\%\;of\;SCV=\frac{AB-AS}{AB}\times 100$$

where, SCV is the cavenging activity; AB is the absorption of the blank (DPPH + methanol); and AS is the absorption of the sample (DPPH + methanol + sample)

## 4. Conclusions

The present work examined the characteristics of jamun seed filler. The filler had a low density (0.59 g/cc) and considerable amounts of cellulose (43.28%), hemicellulose (20.58%), lignin (23.28%), pectin (12.58%), wax (0.98%), and moisture (11.53%) were observed. Due to its lower density, the jamun seed filler can be suitable for fabricating lightweight materials. The FTIR results revealed various absorption bands that correspond to different functional groups, such as cellulose, hemicellulose, and lignin. A crystallinity index of 42.63% was observed. This observation could indicate the degree of crystallinity relative to its amorphous content. Furthermore, the crystallite size was estimated to be 0.398 nm. The particle size analysis reported that the size of jamun filler ranged between 1.98 and 127.66 μm. The diameter range of the fillers was also confirmed using SEM analysis, whereby the ranges were observed between 1 and 40 μm. The EDX spectrum results confirmed the presence of the fundamental elements in the fillers. Moreover, the analysis detected the presence of magnesium (Mg), silicon (Si), aluminum (Al), iron (Fe), and potassium (K). Thermal stability was ensured using a TGA plot, whereby the maximum temperature that the filler was capable of withstanding was 316.5 °C. The endothermal (305 °C) and exothermic (400 °C) peaks were noticed from the DSC results. Due to the lower density of jamun fillers, they can be recommended for automotive applications where weight reduction is crucial to enhance fuel efficiency and reduce emissions. Furthermore, the filler had a considerable amount of cellulose, hemicellulose, and lignin present and exhibited notably high antioxidant activity, suggesting their potential as effective antioxidants. It could be a suitable candidate as a strengthening agent for various lightweight applications in polymeric composites for automotive and household industries.

## Figures and Tables

**Table 1:** Composition of different natural fibers and their unique properties.

Material	Cellulose (%)	Hemicellulose (%)	Lignin (%)	Unique Properties	Ref.
Banana pseudo-stem	62.24	15.23	18.51	High crystallinity, thermal stability up to 250 °C	[10]
Sirisha bark	68.23	13.26	13.25	Stability up to 429 °C, suitable for high-temp applications	[11]
Ficus amplissima root	52.64	10.64	Not reported	Thermal stability at 200 °C, crystallinity index of 39%	[12]
Acacia caesia bark	37.00	20.00	18.00	Thermal degradation at 308 °C	[13]
Champagne cork	Not reported	Not reported	Not reported	Used to replace rubber elastomer in various applications	[14]
Albizia Saman	61.00	15.00	Not reported	High cellulinity supports structural integrity	[15]
Saccharum Bengalense Grass	54.00	32.00	12.00	Good thermal stability, max degradation temp around 340 °C	[16]

**Table 2:** Physicochemical composition of *S. cumini* powder.

Name of the Filler	Density (g/cc)	Cellulose (%)	Hemi Cellulose (%)	Lignin (%)	Pectin (%)	Wax (%)	Ash (%)	Moisture (%)	Ref.
***Syzygium cumini* (L.) Skeels**	***0.59***	***43.28***	***20.58***	***23.28***	***12.58***	***0.98***	***10.76***	***11.53***	***Present work***
Pistacia vera nutshell (PVNS) filler	1.32	47.08	26.56	13.74	-	0.92	4.18	7.52	[27]
Rice husk filler	2.2	23–46	19–24	11–32	-	14–16	-	14	[28]
Walnut shell filler	0.51	25.4	46.6	49.1	-	-	3.6	-	[29]
Peanut shell filler	1.46	44.8	5.6	36.1	-	-	3.8	-	[30]
Coconut shell filler	0.7	26.6	-	29.4	-	-	0.6	8	[31]
Sunflower seed hull filler	0.69	31–51	13–28	20	-	3	2–6	-	[32]
Soybean hulls filler	1.03	20	50	2	30	-	4.3	7	[33]

**Table 3:** FTIR peak allocations in the *S. cumini* powder.

Peak Positions (Wavenumber (cm^−1^))	Allocations
3433	O–H stretching (moisture or hydroxyl groups in cellulose)
2889	C–H stretching (methyl and methylene groups in cellulose and hemicellulose)
2360	Possible atmospheric CO_2_ or triple bonds
1730	C=O stretching of lignin and hemicellulose fractions
1629	water absorption
1465	CH_3_ deformation of lignin
1355	C–H bending
1232	C–O stretching of lignin
1157, 1024	Multiple peaks of C–O–C pyranose ring
920, 862	β-glycosidic linkages between glucose units of cellulose

## Data Availability

No new data were created or analyzed in this study. Data sharing is not applicable to this article.
